# A cross-sectional analysis of dermatology resident research productivity in the United States

**DOI:** 10.1016/j.jdin.2025.09.013

**Published:** 2025-10-09

**Authors:** Matthew Tao, Sean O’Hare, Manvinder Suden, Mary Grace Hash, Frances Case, Alex Nguyen, Harris Bolus, Garrett Dyess, Melodi Javid Whitley

**Affiliations:** a University of South Alabama College of Medicine, Mobile, Alabama; b National Institutes of Health, Bethesda, Maryland; c Edward Via College of Osteopathic Medicine, Auburn, Alabama; d Independent Researcher, Honolulu, Hawaii; e Department of Dermatology, Duke University School of Medicine, Durham, North Carolina

**Keywords:** dermatology, dermatology match, dermatology residency, h-index, medical education, medical student research, research productivity, residency match, residency selection criteria, resident productivity

*To the Editor:* Recent changes to the United States Medical Licensing Examination Step 1 scoring have increased the emphasis on nonexam-based metrics for applicants to dermatology, a highly competitive specialty with a large applicant pool.^1^ Specifically, evaluators increasingly rely on factors such as letters of recommendation, leadership roles, and research.^2^ As programs increasingly value research experience when ranking applicants, understanding factors that influence resident productivity, such as medical school rank, degree type, and postgraduate training year (PGY), is essential. To address these understudied metrics, we conducted a cross-sectional analysis of research productivity among dermatology residents from 2021 to 2023, examining data from 1518 residents across 119 US dermatology programs. Each resident’s medical school affiliation and PGY were obtained from residency program websites. Publication counts and h-indices were extracted using Scopus, Python-based scripts, and manual verification. To address name matching, author profiles underwent manual verification by cross-referencing institutional affiliations and coauthors. Quantitative variables were compared using Mann-Whitney *U* and Kruskal-Wallis tests. Medical schools were stratified into 6 tiers based on the Blue Ridge Institute’s 2023 NIH funding rankings. Graduates from top-ranked medical schools (top 25 in NIH funding) had greater median total publication counts (9.0 vs 4.0; *P* < .001), h-indices (4.0 vs 2.0; *P* < .001), and first-author publications (4.0 vs 2.0; *P* < .001), as shown in Table I. These differences extended to publications in top-tier dermatology journals. Residents with MD degrees exhibited greater research productivity than their DO counterparts, with higher median total publications (7.0 vs 2.5; *P* < .001) and h-indices (3.0 vs 1.0; *P* < .001), corroborating previous reports of degree-associated disparities (Fig 1).^3^ PGY-4 residents had higher h-indices than PGY-2 residents (3.0 vs 2.0; *P* = .001), a finding likely attributable to additional time to accrue citations. Despite this difference in h-index, we observed similar publication counts across PGY classes, which could reflect applicants entering residency with more research, thus minimizing differentials across training years.^4^^,^^5^ It may also suggest that research productivity declines during residency. Notably, the median dermatology resident had zero publications in the top 4 dermatology journals by 2023 impact factor, suggesting that publication growth may be occurring in lower-impact journals. We observed no significant differences in research productivity between allopathic and international medical graduates, which contrasts prior studies reporting greater research productivity among international medical graduates matching into dermatology.^3^ This finding may indicate increasing research engagement among US medical graduates, leading to a convergence in scholarly output between these groups. While research experience is considered highly among residency selection committees, our findings suggest research productivity during residency may be limited. This highlights the importance of institutional support and mentorship in facilitating continued research productivity through residency. Limitations include possible misattribution of publications among residents with common names and reliance on publicly accessible information. Additionally, our cross-sectional design cannot distinguish between work completed before versus during residency, as there is often substantial time that elapses between the initiation of a project and its publication. Future efforts focusing on structured mentorship opportunities and targeted research funding, especially at osteopathic and lower resourced institutions, may help ensure equitable academic opportunities in dermatology.

**Table I:** Research productivity metrics by postgraduate training year class and medical school rank

Category	Total residents	H-index median (IQR)	Total publications median (IQR)	First-author publications median (IQR)	Top 2 journals		Top 4 journals	
					First-author median (IQR)	Total median (IQR)	First-author median (IQR)	Total median (IQR)
Total	1518	3 (1-5)	7 (3-13)	3 (1-7)	0 (0-1)	0 (0-2)	0 (0-1)	0 (0-2)
PGY class								
PGY-2	524	2 (1-4)	7 (3-13)	3 (1-6)	0 (0-1)	0 (0-2)	0 (0-1)	0 (0-2)
PGY-3	501	3 (1-5)	6 (3-13)	3 (1-6)	0 (0-1)	0 (0-2)	0 (0-1)	0 (0-2)
PGY-4	493	3 (1-6)	7 (3-15)	3 (1-6)	0 (0-1)	0 (0-2)	0 (0-1)	0 (0-2)
*P* value		**.0011**	.2728	.5037	.6293	.5332	.5778	.4297
Medical school rank								
1-25	376	4 (2-6)	9 (4-16)	4 (2-7)	0 (0-1)	1 (0-2)	0 (0-1)	1 (0-3)
26-50	319	3 (1-6)	9 (3-17)	4 (2-7)	0 (0-1)	1 (0-3)	0 (0-1)	1 (0-3)
51-75	288	2 (1-4)	6 (3-12)	3 (1-5)	0 (0-1)	0 (0-2)	0 (0-1)	0 (0-2)
76-100	188	2 (1-4)	4 (2-9)	2 (0-4)	0 (0)	0 (0-1)	0 (0)	0 (0-1)
≥101	184	2 (1-4)	6 (2-11)	3 (1-5)	0 (0)	0 (0-1)	0 (0-1)	0 (0-2)
Unranked	163	2 (1-4)	4 (2-9)	2 (0-4)	0 (0)	0 (0-1)	0 (0)	0 (0-1)
*P* value		**<.001**	**<.001**	<**.001**	**<.001**	<**.001**	**<.001**	**<.001**

Medical school tiers were defined by 2023 NIH funding ranking. Top 2 dermatology journals by 2023 impact factor were the Journal of the American Academy of Dermatology and JAMA Dermatology. Top 4 journals included the addition of the British Journal of Dermatology and the American Journal of Clinical Dermatology.

Bold values indicate statistical significance (*P* < .05).

*IQR*, Interquartile range; *PGY*, postgraduate training year.

**Fig 1:**
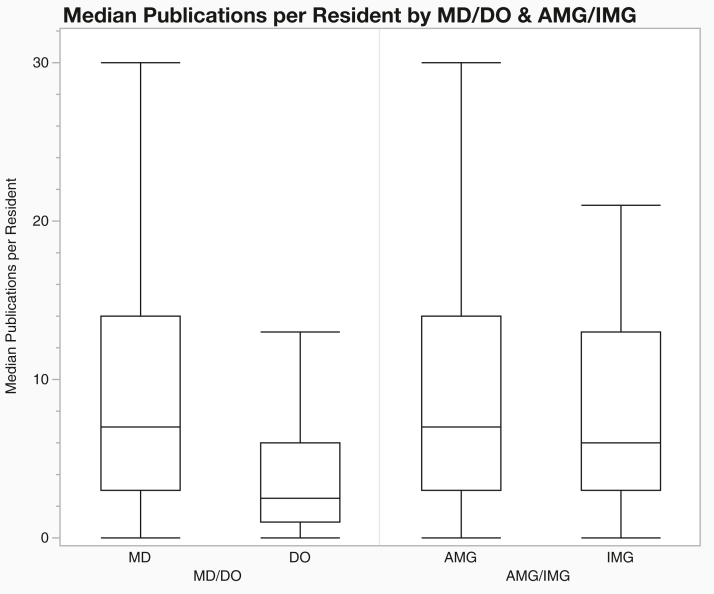
Median publications per resident by degree type (MD vs DO) and medical school origin (AMG vs IMG) among dermatology residents from 2021 to 2023. Box and whisker plots show the median, IQR, and range. Samples sizes were MD (*n* = 1424 residents), DO (*n* = 94 residents), AMG (*n* = 1472 residents), and IMG (*n* = 46 residents). *AMG*, Allopathic medical graduate; *IMG*, international medical graduate; *IQR*, interquartile range.

### Declaration of generative AI and AI-assisted technologies in the writing process

AI was not utilized in manuscript composition.

## Conflicts of interest

None disclosed.
